# Mechanical, Hydrophobic, and Barrier Properties of Nanocomposites of Modified Polypropylene Reinforced with Low-Content Attapulgite

**DOI:** 10.3390/polym14173696

**Published:** 2022-09-05

**Authors:** Chi-Hui Tsou, Rui Zeng, Chih-Yuan Tsou, Jui-Chin Chen, Ya-Li Sun, Zheng-Lu Ma, Manuel Reyes De Guzman, Lian-Jie Tu, Xin-Yuan Tian, Chin-San Wu

**Affiliations:** 1 School of Materials Science and Engineering, Sichuan University of Science and Engineering, Zigong 643000, China; 2 Material Corrosion and Protection Key Laboratory of Sichuan Province, Sichuan University of Science and Engineering, Zigong 643000, China; 3 Sichuan Zhixiangyi Technology Co., Ltd., Chengdu 610051, China; 4 Sichuan Zhirenfa Biotechnology Co., Ltd., Zigong 643000, China; 5 Department of Material and Textile, Asia Eastern University of Science and Technology, New Taipei City 220, Taiwan; 6 Sichuan Vocational College of Chemical Technology, Luzhou 646300, China; 7 Department of Applied Cosmetology, Kao Yuan University, Kaohsiung 82101, Taiwan

**Keywords:** modified polypropylene, attapulgite, nanocomposite, barrier performance, hydrophobicity, compatibility

## Abstract

Attapulgite (ATT) has never been used as a barrier additive in polypropylene (PP). As a filler, ATT should be added in high content to PP. However, that would result in increased costs. Moreover, the compatibility between ATT and the PP matrix is poor due to the lack of functional groups in PP. In this study, carboxylic groups were introduced to PP to form a modified polypropylene (MPP). ATT was purified, and a low content of it was added to MPP to prepare MPP/ATT nanocomposites. The analysis from FTIR indicated that ATT could react with MPP. According to the results of oxygen and water permeability tests, the barrier performance of the nanocomposite was optimal when the ATT content was 0.4%. This great improvement in barrier performance might be ascribed to the following three reasons: (1) The existence of ATT extended the penetration path of O_2_ or H_2_O molecules; (2) O_2_ or H_2_O molecules may be adsorbed and stored in the porous structure of ATT; (3) Most importantly, –COOH of MPP reacted with –OH on the surface of ATT, thereby the inner structure of the nanocomposite was denser, and it was less permeable to molecules. Therefore, nanocomposites prepared by adding ATT to MPP have excellent properties and low cost. They can be used as food packaging materials and for other related applications.

## 1. Introduction

Industrial technology has been continually developed. Alongside this development, the demand for polymers has increased [1,2]. One of the commonly used polymers is polypropylene (PP). Relative to other general-purpose plastics, PP is a colorless, odorless, nontoxic, and translucent solid substance [3]. It has the advantages of small density, excellent processability, strong mechanical properties, low grid, and a wide range of sources [4,5,6,7,8]. PP is widely used in various products such as automotive, electrical appliances, daily necessities and furniture, and packaging [9,10,11,12,13,14,15,16]. However, PP also has some weaknesses; for example, it has low strength and is easy to age. These limitations hamper its varied applications. Therefore, it should be appropriately modified and strengthened through ways such as copolymerization, grafting, blending, or filling [17,18,19].

In recent years, the development of nanomaterials has provided a new method and an idea for the reinforcement and toughening of PP by employing common nano-modified fillers (e.g., kaolin and montmorillonite). At present, the main methods of preparing composites with PP as a matrix are intercalation and direct dispersion. According to the types of fillers added, PP nanocomposites can be roughly divided into the following two categories: one is nanocomposites with layered silicate nanoparticles; the other is nanocomposites with inorganic rigid particles.

Attapulgite (ATT) is nontoxic, and it has a large specific surface area, large reserves, excellent biocompatibility, good adsorption capacity, and is a natural nano-mineral with outstanding performance [20,21]. ATT can be effectively applied in industries and agriculture, and it also has potential applications in many high-tech industries [22,23,24,25,26,27,28]. In the process of modifying polymers with ATT as filler, the interfacial adhesion between ATT and the polymer matrix has a great impact on the properties of the composite materials. In blending ATT and PP, the high specific surface area of ATT has a greater effect on the PP and ATT interface. Additionally, this would enhance the effect of PP. PP without hydroxyl and other functional groups shows hydrophobicity and poor compatibility with ATT. In a PP matrix, ATT easily aggregates, which makes the modification effect unsatisfactory. In this present experiment, PP was modified to form MPP, so that it would have hydroxyl groups, and in turn, the compatibility of ATT with MPP was good enough to achieve a better filling effect.

Chen et al. [29] grafted ATT onto MPP through chemical grafting. When the amount of ATT was 3 wt%, the impact strength and tensile strength of the composite increased by 123 and 23%, respectively, compared with pure PP, and the glass transition temperature decreased. From the crystal structure analysis, the resulting MPP after the addition of ATT had β-crystal morphology, and the relative content of crystals increased by 18.0% [30]. Gao et al. [31] prepared PC/PP/AT ternary nanocomposites by a two-step melt blending method. Their results indicated that PP had a high crystallinity and better toughness, and adding ATT caused PP to have a higher heterogeneous nucleus action. Wang et al. [32] modified ATT with a silane coupling agent and butyl acrylate and prepared nanocomposites with the following different amounts of ATT: 0, 1, 2, 3, 4, and 5%. The results showed that ATT improved the mechanical properties and storage modulus of the nanocomposites. The glass transition temperature of the composites, except for the one with 2% ATT, decreased. Yang et al. [33] prepared nano-sheet composite fillers from ATT and two-dimensional graphene oxide. The tensile strength, Young’s modulus, and storage modulus of the composite increased. This method of adding auxiliary dispersants provided a new idea and a way for a better use of ATT or nanomaterials. Wang et al. [34] prepared a polypropylene/ATT nanocomposite, and the results showed that ATT had a heterogeneous nucleation effect on the PP matrix. ATT increased the non-isothermal crystallization rate and reduced the activation energy of the nanocomposite by 34.6 kJ/mol. A study by Qian et al. [35] found that the tensile strength and impact strength of ATT-filled PP composites increased first and then decreased with increasing ATT content. When the filler amount was 10%, the tensile strength and impact strength were the highest. With an increase in the ATT content, the bending strength and deformation temperature of the composites increased. When PP was filled with the same amount of ATT and CaCO_3_, the comprehensive properties of ATT/PP composites were better. Yang Liu et al. [36] used sulfuric acid to pretreat ATT, and then it was filled into NCC to prepare NCC/ATT nanocomposites. They found that NCC/ATT nanocomposites with 3% ATT achieved increased tensile strength, from 28.5 to 32.85 MPa.

In previous reports, the content of ATT in MPP exceeded 1%. If the content of nanofillers is too high, the cost of preparing nanocomposites increases. Moreover, it is more likely to lead to an uneven dispersion and agglomeration of nanofillers in the matrix. ATT is a porous nanomaterial, and it has a good adsorption capacity for gas or liquid. As such, it can improve the barrier performance of PP. At present, there is no relevant research on the use of ATT as a barrier additive for PP. Our present study introduced carboxylic acid groups to PP to form MPP to improve its interfacial compatibility with ATT and to improve the modification effect. The mechanical, thermal, and barrier properties, as well as the hydrophilicity, of the nanocomposites with varying low content of ATT (0–0.8%) were characterized, with the goal of determining the optimal amount of ATT that should be added. The results obtained from this present study would expand and enrich the application of nanocomposites.

## 2. Experimental Methods

### 2.1. Experimental Materials

PP (5000S) was purchased from Sinopec Yangzi Petrochemical Co., Ltd., Nanjing, China. ATT (HY-P1) was supplied by Shenzhen Haiyang Powder Technology Co., Ltd., Shenzhen, China. Sodium hexametaphosphate (Mn = 611.77 g/mol) was provided by Titan Technology Co., Ltd. (Shanghai, China).

### 2.2. Purified Attapulgite and Preparation of Nanocomposites

**Figure 1:**
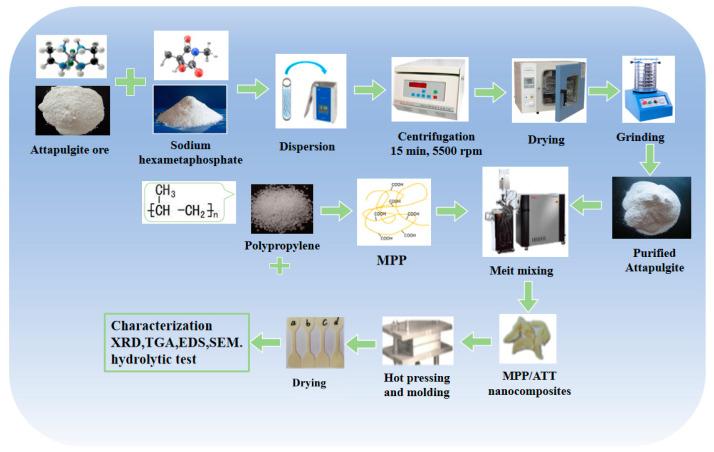
Diagram for the process of fabricating MPP/ATT nanocomposites.

The purification method of ATT was the same as that in a previous study [22]. Figure 1 indicates the process of fabricating MPP/ATT nanocomposites, and the composition of the nanocomposites is shown in Table 1.

### 2.3. Tests and Evaluation of Parameters

#### 2.3.1. Elemental Content Analysis

ATT was scanned using an energy dispersive spectroscopy (EDS), Tescan, Czech Republic.

#### 2.3.2. Fourier Transform Infrared Spectroscopy

The spectra of nanocomposites were measured using a Fourier transmission infrared spectrometer (Nicolet 6700 FTIR). The measurement range was 4000–400 cm^−1^, and it was performed at a resolution of 1 cm^−1^. The nanocomposites were formed into a powder, and then mixed with KBr and pressed into circular samples for testing.

#### 2.3.3. Mechanical Properties

Samples of MPP and MPP/ATT nanocomposites were prepared following ASTM-D638 test standard and tested to determine the tensile properties by using a tensile testing machine (speed = 50 mm/min^−^^1^). Five to seven samples for each nanocomposite were tested for tensile properties, and the average value was calculated.

#### 2.3.4. X-ray Diffraction

X-ray diffractometry was used. The instrument was from Bruker, Germany (D8 Advance). It was operated at a voltage of 40 KV.

#### 2.3.5. Morphology Characterization

Scanning electron microscopy (SEM) was applied to obtain the morphology of the nanocomposites. The tensile fractured surface of all samples was scanned by SEM (Tescan, Czech Republic).

#### 2.3.6. Thermal Analysis

Differential scanning calorimetry (DSC-200 F3 calorimeter) was from Netzsch, Germany. The nanocomposites were subjected to a second heating at a rate of 10 °C/min from room temperature to 180 °C and held at that high temperature for 5 min. Then, they were cooled down to room temperature at a rate of 10 °C/min to obtain crystallization peaks. The equation for calculating the MPP crystallinity was as follows:

(1) $$X_{C}=\left(\frac{△H}{△H_{0}\times wt\%}\right)\times 100\%$$

In Equation (1), *Xc* = degree of crystallinity, *wt*% = percentage of MPP in the sample, ∆*H* was the enthalpy of fusion for MPP and its nanocomposites, and Δ*H*_0_ the enthalpy of fusion for 100% PP polymer [37].

#### 2.3.7. Thermogravimetric Analysis

Thermal gravimetric analysis (TGA, Model HTG-1, Beijing Hengjiu Experimental Equipment Co., Ltd., Beijing, China) was applied to test the samples. The nanocomposites were heated from room temperature to 750 °C under nitrogen environment (flow rate = 70 mL/min) at a heating rate of 10 °C/min,

#### 2.3.8. Water Vapor Barrier Properties

A water vapor permeability (WVP) tester was operated at 25 °C, and the humidity difference on both sides of the nanocomposites reached 90%.

#### 2.3.9. Oxygen Barrier Properties

The oxygen transmission rate (OTR) in MPP and MPP/ATT nanocomposites was measured with a gas permeability meter (Labthink VAC-V2, Jinan, China). It was operated at 23 °C and 50% humidity.

#### 2.3.10. Water Absorption

All nanocomposites were immersed in deionized water for 48 h. They were then weighed, and the water absorption was calculated as follows:

(2) $$W_{A}=\frac{W_{0}-W}{W_{0}}\times 100\%$$

In Equation (2), *W*_A_ represented the water absorption, *W* was the initial weight of the nanocomposites, and *W*_0_ the weight of the sample after soaking [38].

#### 2.3.11. Contact Angle Test

A contact angle machine was used to evaluate contact angles (CA). First, all nanocomposites were dried in an oven at 110 °C for 12 h. Then, deionized water was dropped using a pipette onto the nanocomposite samples.

## 3. Results and Discussion

### 3.1. Elemental Content Analysis

**Figure 2:**
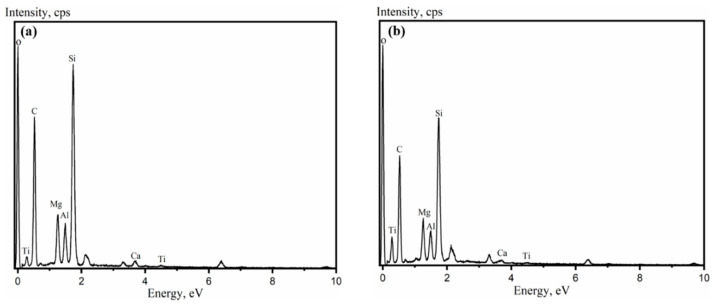
Energy dispersion spectroscopy of ATT: (**a**) unpurified ATT, (**b**) purified ATT.

Data on EDS of unpurified ATT and purified ATT are shown in Figure 2. Table 2 lists the elemental composition of both ATT. Purified ATT has a higher composition of C, but a slightly lower composition of each other elements. This may be caused by the removal of a large number of impurities from the unpurified ATT.

### 3.2. Fourier Transform Infrared Spectra

**Figure 3:**
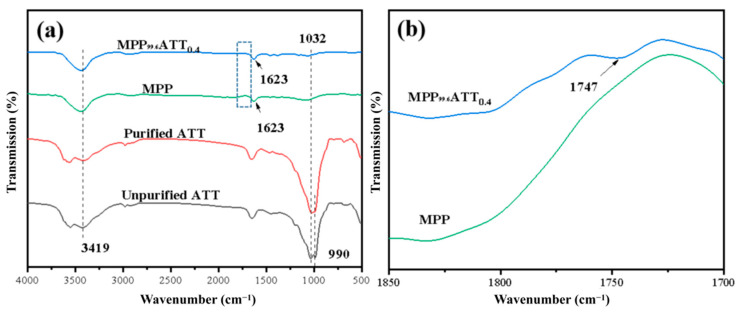
FTIR spectra: (**a**) MPP_99.6_ATT_0.4_, MPP, purified ATT, and unpurified ATT for wavenumbers 500–4000 cm^−1^; (**b**) MPP_99.6_ATT_0.4_ and MPP for wavenumbers 1700–1850 cm^−1^.

Figure 3 represents the FTIR spectra of unpurified ATT, purified ATT, MPP, and MPP_99.6_ATT_0.4_. In the FTIR of unpurified ATT, symmetrical and asymmetrical stretching vibration peaks in the 900–1100 cm^−1^ region refer to the silicon-oxygen bond. The vibration peak at about 3419 cm^−1^ denotes the silanol structure, indicating that ATT has a large number of hydroxyl groups [39]. Compared with unmodified ATT, purified ATT exhibits a higher intensity of absorption peak, which was probably caused by the removal of some impurities such as quartz. In the FTIR of MPP, the obvious peak that occurs in the band of about 3435 cm^−1^ corresponds to the hydroxyl group of MPP [40,41]. The characteristic peak near 1623 cm^−1^ is the bending vibration peak of –CH_2_– in the infrared spectrum of MPP_99.6_ATT_0.4_ [42]. Symmetrical and asymmetrical telescopic vibration peaks in the 900–1100 cm^−1^ region are equivalent to the silicon-oxygen bond absorption peaks. In addition, a new feature peak is observed at 1747 cm^−1^ in the enlarged local plot of Figure 3b, and the characteristic peak is due to the C=O peak in the ester group [17]. The characteristic peak is caused by the reaction between –OH on the ATT surface and –COOH on MPP. FTIR results show that ATT can react with MPP, and this increases the reinforcing effect of nanofillers on polymers [43].

### 3.3. Tensile Strength

**Figure 4:**
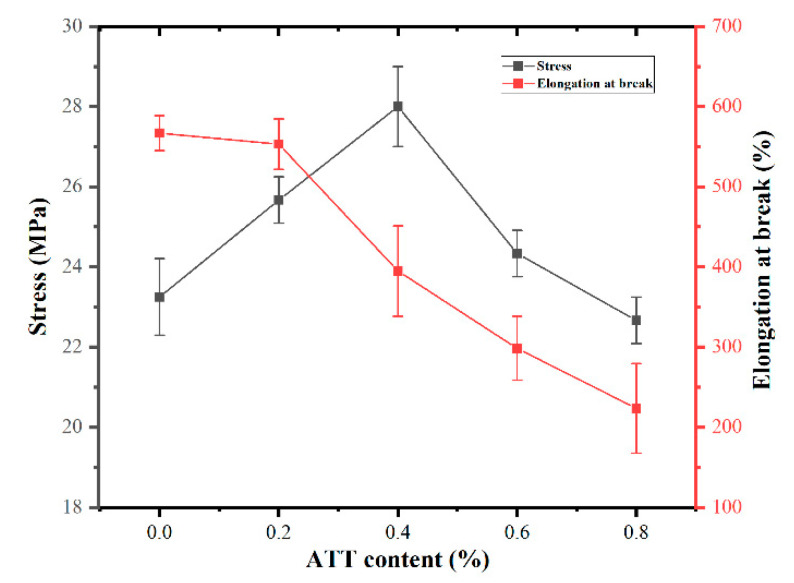
Stress and elongation at break of MPP/ATT nanocomposites containing different amounts of ATT.

The tensile properties of all samples with various amounts of purified ATT were obtained. Figure 4 shows the difference in the tensile properties of MPP and MPP/ATT nanocomposites with different ATT content. Compared with the tensile strength of neat MPP, that of the MPP/ATT nanocomposites is remarkably enhanced. The tensile strength is improved with an increase in the ATT content from 0 to 0.4%. The tensile strength reaches its maximum value when ATT is 0.4%, demonstrating that ATT could enhance the tensile properties of the polymer matrix. This may also be due to the reaction between the –COOH in MPP and the hydroxyl group in ATT, leading to improvement in the tensile strength of the nanocomposites [44,45]. However, when the nanofiller is ≥0.6%, the tensile strength decreases [46]. This may be due to the fact that ATT does not disperse easily in the substrate, thereby aggregation is increased. The poor compatibility between the nanofiller and MPP results in stress concentration in the nanocomposites and, in turn, in decreased mechanical strength [47,48].

### 3.4. X-ray Diffraction Patterns

**Figure 5:**
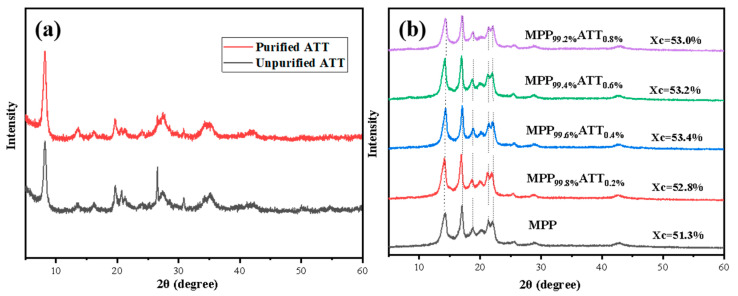
Diffraction peak intensity diagram. (**a**) purified and unpurified ATT; (**b**) MPP and MPP/ATT nanocomposites with different amounts of ATT.

Figure 5a displays XRD patterns for ATT before and after purification. The peak of purified ATT is higher and sharper, probably because of the great reduction in impurities. In Figure 5b, MPP has strong peaks around 14.3° and 17.2°. The characteristic peaks of the nanocomposite materials are hardly shifted, suggesting that adding purified ATT may not affect the crystal planes of MPP. There is no major change in the crystalline morphology of MPP, showing that the compatibility between MPP and ATT is good [49]. Relative to MPP, the nanocomposites have increased crystallinity. Additionally, MPP_99.6_ATT_0.4_ has the largest crystallinity. It might be ascribed to the heterogeneous nucleation of ATT that improves the crystallinity [50].

### 3.5. Morphological Images

**Figure 6:**
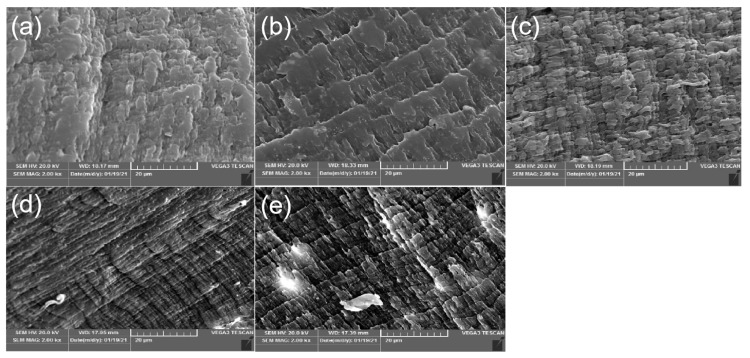
Surface SEM micrographs of fractured MPP/ATT nanocomposites with various amounts of ATT: (**a**) MPP; (**b**) MPP_99.8_ATT_0.2_; (**c**) MPP_99.6_ATT_0.4_; (**d**) MPP_99.4_ATT_0.6_; (**e**) MPP_99.2_ATT_0.8_.

Figure 6 shows the morphological SEM images for the tensile section of MPP and MPP/ATT nanocomposites. It is observed that the tensile section morphology of the nanocomposite with 0.4% ATT is very similar to that of MPP and MPP with an ATT content of 0.2%. This may be because both 0.2 and 0.4% ATT can be uniformly distributed in MPP, and nanomaterials with a high specific surface area can have a good interaction with the matrix despite tensile fracture. This is consistent with the results of tensile properties. The two compositions of 0.2 and 0.4% ATT greatly enhance the tensile strength of the nanocomposites. Due to the tight connection between ATT and MPP, the fracture surface appears to have a structure of uneven surface [51]. However, the addition of ATT ≥ 0.6 results in an uneven distribution of nanoparticles. This may be because of the poor interfacial adhesion between the nanofillers and the MPP matrix. The result is a decrease in the mechanical properties of MPP/ATT, and the tensile section of the nanocomposite shows a unidirectional fiber-like structure [52,53]. It may be a more brittle fracture surface caused by the uneven dispersion of nanofillers.

### 3.6. Thermal Stability Analysis

**Figure 7:**
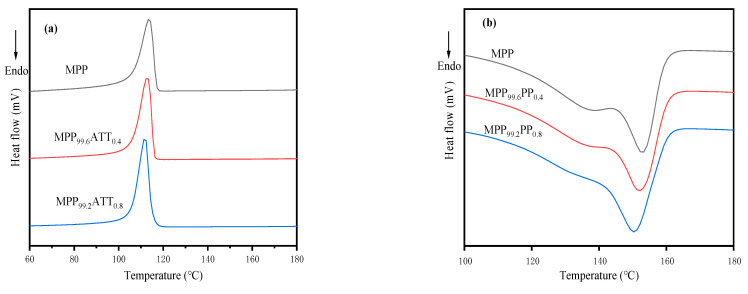
Differential scanning calorimetry for MPP and MPP/ATT nanocomposites: (**a**) first cooling curves; (**b**) second heating curves.

Figure 7 shows the first cooling and secondary heating curves for MPP, MPP_99.6_ATT_0.4_, and MPP_99.2_ATT_0.8_ nanocomposite materials, and the data for representative samples of MPP/ATT nanocomposite materials are tabulated in Table 3. *Tm* and *Xc* of neat MPP are 150.3 °C and 43.40%. The nanocomposites containing ATT show a decreasing trend of *Tm* with increasing ATT content. The reason for the slight decrease in Tm may be due to that ATT makes the crystal size of MPP smaller, so that the nanocomposite melts early during the heating process [54]. On the other hand, *Xc* for nanocomposite materials reveals an increasing trend. It might be ascribed to the heterogeneous nucleation of ATT that improves the crystallinity. Figure 7 shows that the crystallization peak for the MPP nanocomposite materials becomes sharp, indicating that the existence of ATT reduces the crystallization time and greatly improves the crystallization rate.

### 3.7. Thermogravimetric Analysis

**Figure 8:**
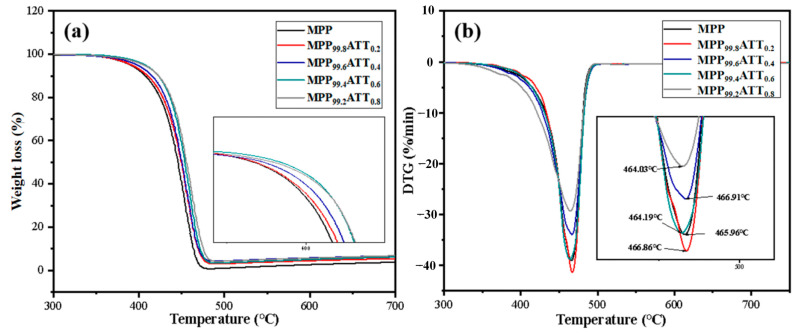
Differential and thermal gravimetric analysis for MPP and MPP/ATT nanocomposites: (**a**) weight loss (%) vs. temperature; (**b**) derivative mass (DTG) vs. temperature.

Figure 8 shows the TGA and DTG curves for MPP and its nanocomposites. Table 4 lists TGA and DTG data taken from Figure 8. *T_onset_* pertains to the beginning of sample degradation, while *T_end_* to the ending of sample degradation, and T_d_ indicates the peaks from DTG. The data clearly shows that adding ATT could increase the *T_onset_*, *T_end_*, and *T_d_* of the nanocomposites. This can be primarily attributed to the excellent heat resistance of ATT and the dense structure of the nanocomposites. Alternatively, carboxyl from MPP and hydroxyl from ATT undergo chemical reactions. Therefore, more energy is required for the decomposition during the heating process, so the thermal stability of the nanocomposites increases [55]. When the ATT content is ≥0.6%, the *T_d_* of the samples is reduced, which may be due to the ATT agglomeration in the MPP matrix. Incompatibility between the matrix and the nanofillers sets in and, in turn, affects thermal degradation [56,57].

### 3.8. Analysis of Water Vapor Barrier Properties

**Figure 9:**
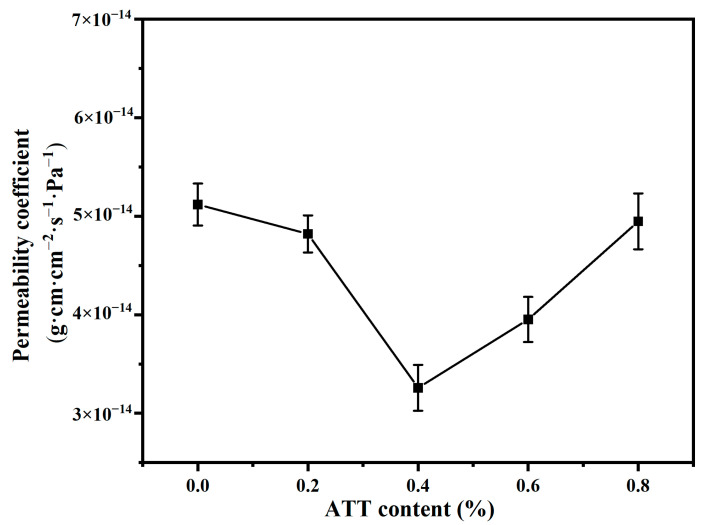
Variation in water permeability coefficients for MPP and MPP/ATT nanocomposite materials.

Figure 9 shows the variation in water permeability for MPP and MPP/ATT nanocomposite materials. With the addition of ATT nanomaterials, the water permeability coefficient tends to decrease first and then increase. When the ATT content is 0.4%, the water vapor permeability coefficient is the lowest. The reason for the decrease in the permeability coefficient may be because the addition of ATT increases the permeability path for the water molecules [58,59]. Another reason is that when MPP reacts with ATT, the internal structure of the nanocomposite becomes tight, causing more difficulty for the water molecules to penetrate. When the ATT addition is between 0.6 and 0.8%, the water permeability coefficient of the nanocomposites increases, which may be due to the excessive addition of ATT. Consequently, the dispersion of ATT in the matrix becomes poor, and agglomeration of ATT occurs, resulting in defects in the interior of the nanocomposites, allowing the water molecules to pass through the gap.

### 3.9. Analysis of Oxygen Barrier Performance

**Figure 10:**
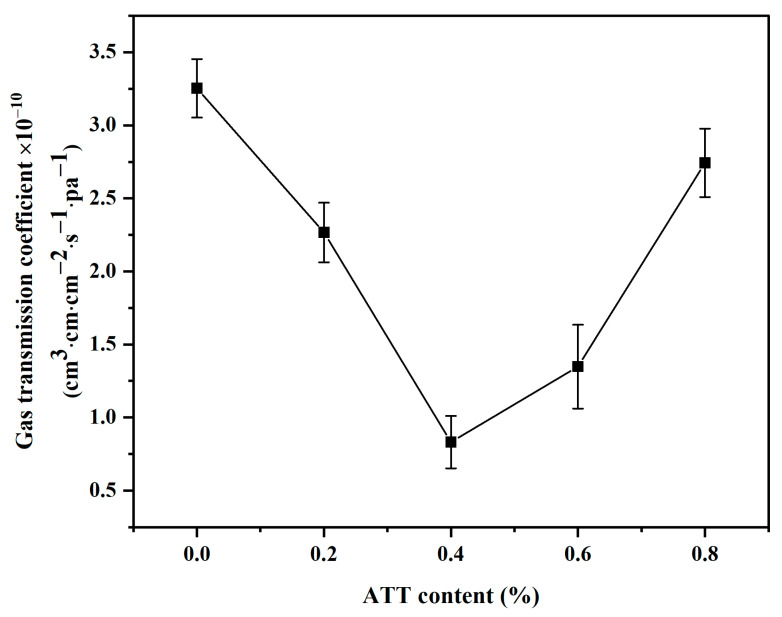
Gas transmission coefficients for MPP and MPP/ATT nanocomposites.

We see in Figure 10 that the oxygen permeability coefficient (OPC) for MPP is 3.183 × 10^−10^ cm^3^ cm cm^−2^ s^−1^ Pa^−1^. As the content of ATT increases, the oxygen permeability shows a trend of first decreasing and then increasing. The OPC of all nanocomposites is lower than that of pure MPP. When the added ATT nanofiller is 0.4%, OPC reaches the minimum value. However, when the nanofiller is ≥0.6%, OPC reverts to increasing. The reason is that the high ATT content causes an uneven dispersion and incompatible defects. Therefore, the arrangement of ATT in the matrix becomes chaotic, and the structure of the nanocomposites is destroyed [53].

The trend in Figure 10 is very similar to the results of water permeability coefficients in Figure 9. ATT plays an important role in the oxygen and water vapor barrier properties of MPP. This may be due to the following three reasons: (1) The addition of ATT nanomaterials would extend the path for the passage of water molecules; (2) ATT has a porous structure, which can adsorb water or oxygen molecules into the internal pores of ATT; (3) –COOH of MPP reacts with –OH on the surface of ATT. The MPP and ATT have good interfacial compatibility, which makes the internal structure of the nanocomposite material more compact, and the extent of the permeation of H_2_O or O_2_ is less [60].

**Figure 11:**
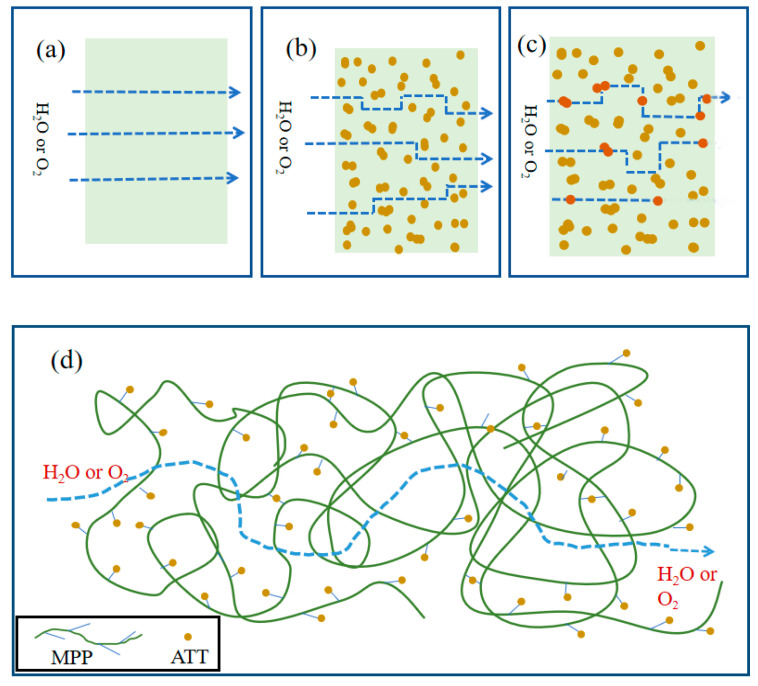
Schematic diagram of gas molecules diffusing through MPP or MPP/ATT nanocomposite: (**a**) diffusion path through MPP; (**b**) diffusion path around MPP/ATT nanocomposite; (**c**) diffusion path within and about MPP/ATT nanocomposite; (**d**) diffusion of water and gas molecules through nanocomposite.

Figure 11 shows the path of H_2_O or O_2_ molecules through the nanocomposite. Solid dots represent ATT dispersed in the nanocomposite. O_2_ or H_2_O molecules would directly pass through a sample of MPP (Figure 11a). The path of water or oxygen molecules through the nanocomposites may be described in two ways. One way is that the molecules may not pass through the ATT nanofillers and move around them (Figure 11b). Another way is that the molecules may penetrate and linger within ATT and eventually exit from there (Figure 11c). In the nanocomposite, the –COOH in MPP reacts with –OH on the ATT surface, and then the MPP molecular chains become more tightly connected, causing a reduction in the transmittance of H_2_O or O_2_ molecules (Figure 11d).

### 3.10. Water Absorption Analysis

**Figure 12:**
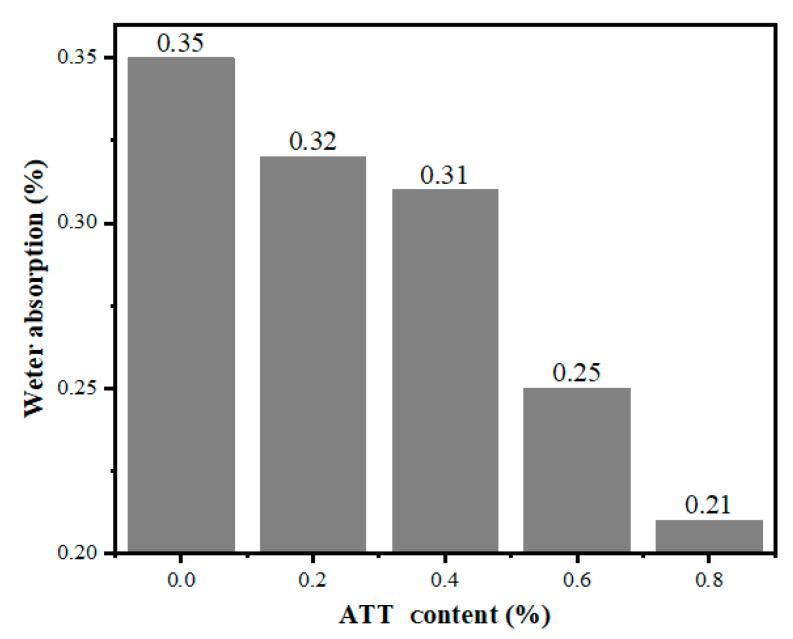
Water absorption data for MPP and ATT/MPP nanocomposites.

Figure 12 presents data on the water absorption in MPP/ATT nanocomposites. The results indicate that the water absorption rate for MPP is high. With increasing ATT content, the nanocomposite materials show a trend of decreasing water absorption rates. ATT has a good water absorption performance owing to its porous and rod-like structure. However, when the ATT–OH reacts with the MPP–COOH, the nanocomposite forms a tighter mesh structure. The MPP tightly wraps the water-absorbing ATT inside, and this reduces the water absorption of the nanocomposite.

### 3.11. Contact Angle Data

**Figure 13:**
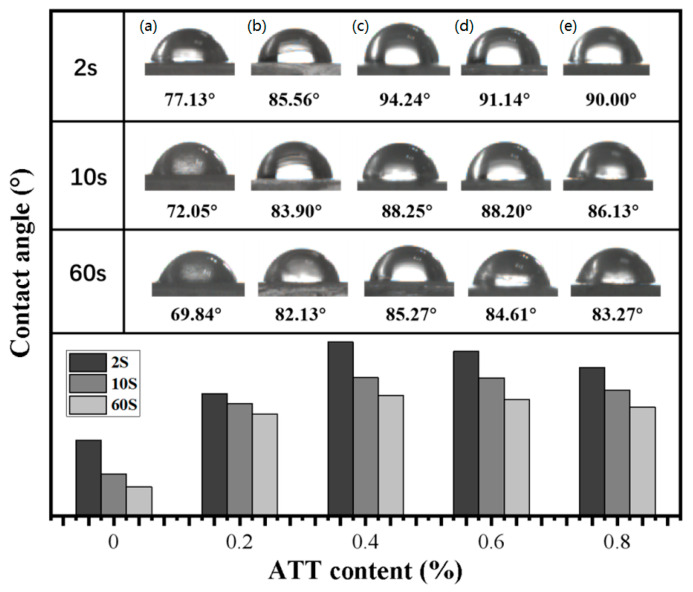
Contact angle data: (**a**) MPP; (**b**) MPP_99.8_ATT_0.2_; (**c**) MPP_99.6_ATT_0.4_; (**d**) MPP_99.4_ATT_0.6_; (**e**) MPP_99.2_ATT_0.8_.

Figure 13 shows the results of contact angle tests, in which water droplets are made to touch the surface of MPP and MPP/ATT nanocomposite samples for 2, 10, and 60 s. With an increase in the ATT content, the contact angles of the nanocomposites first increase and then decrease. When the ATT content is 0.4%, the contact angle is the largest. This may be because ATT nanomaterials are widely and uniformly dispersed in the matrix. As such, the nanomaterials are more compact in structure and show a hydrophobic state. This is in agreement with the results of water permeability coefficients and oxygen permeability tests. When ATT > 0.4%, it shows an uneven dispersion in the matrix. The structure of the nanocomposites has more defects, and the water molecules are more likely to enter the nanocomposites. In addition, ATT itself is a porous water-absorbing material, and when the ATT content is too much, the hydrophilicity of the nanocomposites may increase. The results show that the contact angle of each nanocomposite is greater than that of MPP. The results show that the hydrophobicity of the nanocomposite can be improved with the optimal amount of ATT.

According to the analysis of data on the contact angle, barrier properties, and tensile properties, incorporating 0.4% ATT into MPP can make the nanocomposite film have the most compact structure, the highest hydrophobicity, and the best tensile strength. This nanocomposite film with the optimum amount of added ATT has the potential possibilities to be used as a packaging material.

## 4. Conclusions

The introduction of ATT significantly improved the performance of MPP. The presence of ATT improved the barrier efficiency of the nanocomposite. Not only ATT could extend the path for oxygen or water molecules in the nanocomposite, but also ATT itself could hold the molecules within its storage for some time. Moreover, MPP could have a chemical reaction with ATT, which would make its internal structure more compact. The results showed that when the ATT content was 0.4%, the nanocomposite demonstrated the best mechanical, thermal, and barrier properties. Hence, it has potential applications and can be further explored in many fields.

## Figures and Tables

**Table 1:** Compositions of MPP and MPP/ATT nanocomposites.

Sample	MPP (%)	ATT (%)
MPP	100	0
MPP_99.8_ATT_0.2_	99.8	0.2
MPP_99.6_ATT_0.4_	99.6	0.4
MPP_99.4_ATT_0.6_	99.4	0.6
MPP_99.2_ATT_0.8_	99.2	0.8

**Table 2:** Elemental compositions of unpurified and purified of ATT.

Sample	Unpurified ATT	Purified ATT
O, *wt*%	52.21	46.48
C, *wt*%	14.25	29.09
Si, *wt*%	17.95	12.23
Mg, *wt*%	6.00	4.41
Al, *wt*%	4.24	3.09
Ca, *wt*%	1.13	0.45
Ti, *wt*%	0.29	0.21

**Table 3:** Data on melting and crystallization.

Sample	*Tc* (°C)	*Tm* (°C)	∆*Hm* (Jg^−1^)	*Xc* (%)
MPP	113.3	153.3	73.13	43.40
MPP_99.6_ATT_0.4_	112.5	152.3	75.63	45.06
MPP_99.2_ATT_0.8_	111.4	150.3	77.37	46.29
Standard deviation	0.78	1.25	1.74	1.18

**Table 4:** Thermogravimetric/differential analysis of MPP/ATT nanocomposites.

Sample	*T_onset_* (°C)	*T_end_* (°C)	*T_d_* from DTG (°C)
MPP	390.21	477.86	465.96
MPP_99.8_ATT_0.2_	391.53	484.51	466.91
MPP_99.6_ATT_0.4_	397.89	483.06	466.86
MPP_99.4_ATT_0.6_	407.39	484.09	464.19
MPP_99.2_ATT_0.8_	405.85	487.63	464.03

## Data Availability

Data sharing not applicable.
